# Comparison of Ground Release and Drone-Mediated Aerial Release of *Aedes aegypti* Sterile Males in Southern Mexico: Efficacy and Challenges

**DOI:** 10.3390/insects13040347

**Published:** 2022-03-31

**Authors:** Carlos F. Marina, Pablo Liedo, J. Guillermo Bond, Adriana R. Osorio, Javier Valle, Roberto Angulo-Kladt, Yeudiel Gómez-Simuta, Ildefonso Fernández-Salas, Ariane Dor, Trevor Williams

**Affiliations:** 1 Centro Regional de Investigación en Salud Pública—Instituto Nacional de Salud Pública, Tapachula 30700, Chiapas, Mexico; gbond@insp.mx (J.G.B.); rosorio.adriana@hotmail.com (A.R.O.); ildefonso.fernandez@uanl.edu.mx (I.F.-S.); 2 El Colegio de la Frontera Sur (ECOSUR), Unidad Tapachula, Tapachula 30700, Chiapas, Mexico; pliedo@ecosur.mx (P.L.); jvalle@ecosur.mx (J.V.); ador@ecosur.mx (A.D.); 3 Servicios Mubarqui, Ciudad Victoria 87040, Tamaulipas, Mexico; complexdynamics@protonmail.com; 4 Programa Moscas de la Fruta (SADER-IICA), Metapa de Domínguez 30860, Chiapas, Mexico; yeudielgomez@prodigy.net.mx; 5 Facultad de Ciencias Biológicas, Universidad Autónoma de Nuevo León (UANL), San Nicolás de los Garza 66450, Nuevo León, Mexico; 6 Consejo Nacional de Ciencia y Tecnologiá (Investigadora por México CONACYT), El Colegio de la Frontera Sur, Unidad Tapachula, Tapachula 30700, Chiapas, Mexico; 7 Instituto de Ecología AC (INECOL), Xalapa 91073, Veracruz, Mexico

**Keywords:** sterile insect technique, release techniques, recapture rates, vector control, release time, physical injury

## Abstract

**Simple Summary:**

Diseases transmitted by the mosquito *Aedes aegypti*, such as dengue, chikungunya and Zika, affect millions of people in tropical and subtropical regions of the world. The sterile insect technique (SIT) is a safe and environmentally benign method of population suppression that could be applied to reduce mosquito-transmitted disease. SIT involves the release of large numbers of sterile male insects that then compete with wild males in mating with females. The females that mate with sterile males do not produce viable offspring. To test this technique within a pilot-scale trial in a village in southern Mexico, we compared two methods for the weekly release of large numbers (approximately 85,000 males/week) of sterile males that were marked with colored powders for later identification. The number of sterile males captured in traps placed in and around houses was higher (average 5.1 males/trap/week) for sterile males released at ground level by technicians walking through the streets of the village. In contrast, traps captured an average of 2.6 males/trap/week when males were released from a small drone aircraft that flew over the village. The males released from the drone may have suffered from chilling, compaction and physical injury during the release procedure. However, the use of the drone resulted in a less aggregated distribution, was markedly quicker, released males over a larger area and required fewer technicians than ground-based releases. Some village residents also reported discomfort from the presence of large numbers of male mosquitoes when released at ground level. Future studies should focus on modifications to the handling and transport of sterile males and the design of drone release containers to avoid injury to sterile mosquitoes and to improve the efficiency of aerial releases for SIT-based suppression of *Ae. aegypti* in Mexico.

**Abstract:**

Sterile males of *Aedes aegypti* were released once a week for 8 weeks to evaluate the dispersal efficiency of ground and aerial drone release methods in a rural village of 26 Ha in southern Mexico. Indoor and outdoor BG-Sentinel traps were placed in 13–16 houses distributed throughout the village. The BG traps were activated 48 h after the release of the sterile males and functioned for a 24 h period following each release. Over the 8-week period of simultaneous ground and aerial releases, an average of 85,117 ± 6457 sterile males/week were released at ground level and 86,724 ± 6474 sterile males/week were released using an aerial drone. The ground release method resulted in higher numbers of captured males (mean = 5.1 ± 1.4, range 1.1–15.7 sterile males/trap) compared with the aerial release method (mean = 2.6 ± 0.8, range 0.5–7.3 sterile males/trap) (*p* < 0.05). Similarly, the prevalence of traps that captured at least one sterile male was significantly higher for ground release compared to the aerial release method (*p* < 0.01). The lower numbers of sterile males captured in the aerial release method could be due to mortality or physical injury caused by the chilling process for immobilization, or the compaction of these insects during transport and release. However, aerial releases by a two-person team distributed insects over the entire village in just 20 min, compared to ~90 min of work for a five-person team during the ground release method. Ground release also resulted in higher aggregations of males and some villagers reported feeling discomfort from the presence of large numbers of mosquitoes in and around their houses. We conclude that modifications to the handling and transport of sterile males and the design of containers used to store males are required to avoid injury and to improve the efficiency of aerial releases for area-wide SIT-based population suppression programs targeted at mosquito vectors of human disease.

## 1. Introduction

*Aedes aegypti* is the main vector of dengue, chikungunya, Zika and yellow fever viruses in the Americas [1,2,3,4]. The invasive Asian tiger mosquito, *Ae. albopictus* also participates in the transmission of these viruses, as a secondary vector, and is also widely distributed [5]. The control of *Aedes* spp. populations mainly involves the elimination of empty containers, trash and discarded vehicle tires that are used as oviposition sites for the development of immature mosquitoes [6]. Domestic and peridomestic water containers are also treated with insecticides to prevent the development of larvae [1,7]. Additionally, during dengue outbreaks, the residual spraying of houses and nebulization of streets and surrounding areas with pyrethroid and carbamate insecticides is performed to try to further reduce adult populations [8,9].

These vector control strategies have not achieved the desired level of success due to the low penetration of insecticides into houses during nebulization [10], increasing mosquito resistance to insecticides [9,11,12,13,14], operational failures in removing oviposition sites and insufficient coverage of control activities in affected areas [1]. As there are currently no effective vaccines for the main vector-borne diseases [9,15], it is necessary to implement innovative and effective vector suppression strategies that can be incorporated into integrated programs of vector control alongside other control activities.

The sterile insect technique (SIT) is a species-specific, non-polluting, environmentally benign alternative method for vector control [16,17]. The effective implementation of SIT-based control programs requires an area-wide integrated approach that combines the release of sterile males with conventional mosquito control measures [17,18,19,20,21]. Several components are required for the successful implementation of an SIT program [16,17]. Quality control procedures for the mass-rearing, sterilization, transport and systematic release of large numbers of sterile males are required so that these males can effectively outcompete the wild fertile males to mate with wild females [16,20,21].

Following sterilization and transport to the field site, sterile males can be released by means of ground releases in the pupal [22] or adult stages [23,24]. Adult males can also be dispersed by aerial releases [25,26]. Sterile males are often immobilized by chilling to reduce movement and physical injury, and to increase efficiency during transport and release [27]. However, the survival and flight capacity of males can decrease following exposure to low temperatures, or by the physical compaction of males [27,28,29,30]. These issues do not usually arise in males that are not physically compacted or exposed to low temperatures [22,23,24].

The collection of baseline entomological data is required prior to SIT-based interventions to assess the density, distribution and dynamics of the target vector population and the risk of vector displacement by secondary vector species, if present in the study area [16,31,32,33]. Recently, two spatially separate rural village communities 4 km apart with similar environmental and social conditions were selected to validate the efficacy of an SIT program for the suppression of *Ae. aegypti* in Mexico within the scope of a pilot project [34]. In this project, procedures for mass-rearing were developed [35], the optimal sterilizing doses of irradiation for *Ae. aegypti* and *Ae. albopictus* were determined [36], and the competitiveness of sterilized vs. wild males was compared under laboratory and field cage conditions [37]. Community engagement activities were also carried out in both villages to inform the local community of the aims and the activities of the SIT-based pilot project and to obtain the consent of the villagers that were willing to participate in the trial. A three-year baseline study on *Ae. aegypti* and *Ae. albopictus* population fluctuations in and around both pilot villages was then performed [34].

An evaluation of potential release methods is required to compare the efficiency of each method, to identify potential limitations and how these could be mitigated or overcome given the environmental and ecological conditions prevailing in southern Mexico. The aim of the present study, therefore, was to compare the dispersal of sterile males of *Ae. aegypti* released both at ground level and using an airborne drone in a rural village community, as a pilot study prior to the implementation of an area-wide integrated vector suppression program involving SIT in southern Mexico.

## 2. Materials and Methods

### 2.1. Ethical Issues

Starting in 2017, we performed community engagement activities related to an SIT-based research project centered around the villages of Hidalgo and Río Florido in Chiapas, southern Mexico. Prior to the release of sterile males for the present study, we organized a series of meetings, assemblies and workshops with the local municipal authorities, health authorities and residents in which we demonstrated the innocuous nature of male mosquitoes. We also demonstrated drone flight patterns and how the activity of the drone would not affect the privacy or health of villagers. We also clarified that the release of irradiated sterile males would not cause any harm to the environment, and posed no health risks to local inhabitants. The study only began when we had obtained the consent and authorization of all stakeholders including the Mexican Federal Disease Control Authority (CENAPRECE), State and municipal health authorities, the local ejido authorities (agricultural landowners) and village inhabitants.

### 2.2. Study Site, Mosquito Strain, Mass-Rearing and Sterilization

**Figure 1:**
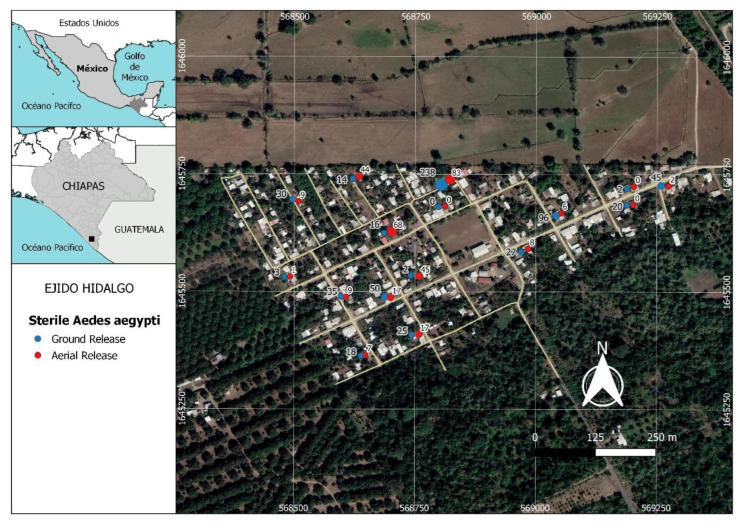
Distribution of sampling points used to recapture sterile males of *Aedes aegypti* released in Hidalgo village in Chiapas, southern Mexico. Each pair of points (red and blue) represents the results from one trap in one house. The blue points and numbers on the left side correspond to the recaptures of males released at ground level. The red points and the numbers to the right side represent recaptures of males released by aerial drone. Values represent the total number of sterile males recaptured in each trap during the 8 weeks of releases using both ground and aerial methods.

The study was carried out in Hidalgo rural village (14°53′4′′ N, 92°21′28′′ W), located in an area of 26 hectares, with 184 inhabited houses and 697 inhabitants, at a distance of 18.4 km from the Pacific Ocean and approximately 14 km from the city of Tapachula, Chiapas State, in southern Mexico (Figure 1). The climate in this area is warm subhumid with a dry and a rainy season [38]. Rains occur from April to November, followed by a dry season from November to April; the prevailing winds are westerly. 

The *Ae. aegypti* strain used in this study was started using eggs collected from 12 urban localities in Chiapas State, Mexico, that were reared and subsequently maintained under laboratory conditions [35,36]. Mass production of this *Ae. aegypti* strain was carried out in the field station facilities of the Centro Regional de Investigación en Salud Pública (CRISP/INSP), located on the edge of Río Florido village at a distance of ~5 km from Hidalgo village [37]. Adult mosquitoes were maintained at 26 ± 2 °C, 80% relative humidity, and a photoperiod of 12:12 h (light: dark) in acrylic cages 30 × 30 × 30 cm (Bug Dorm 1, Taichung, Taiwan, China) at a ratio of 3:1 (females: males) and were fed ad libitum with 10% sucrose solution on a cotton strip. Females were provided with lamb’s blood containing 3.8% sodium citrate as an anticoagulant. Blood meals were offered twice a week, beginning at 5 days post-emergence by means of plastic artificial feeders. Lamb’s blood was never more than 7 days old.

Larvae for *Ae. aegypti* male production were reared at a density of 3 larvae/mL in 61 × 40 × 7 cm plastic trays containing a 3 L volume of dechlorinated water and were fed with powdered Laboratory Rodent Diet (LabDiet 5001, PMI Nutrition International LCC, St. Louis, MO, USA), as described previously [35]. Male pupae were sexed based on their body size by means of plate separators (John H. Hock, Model 5412, Gainesville, FL, USA). Following separation, the genital lobe was visually checked using a stereomicroscope (Stemi 508, Carl Zeiss, Oberkochen, Germany). Female pupae were discarded. Batches of approximately two thousand male pupae were irradiated between 24–36 h prior to the emergence of adults, at the MOSCAFRUT (SENASICA—IICA) facility, Metapa, Chiapas, Mexico. A dry storage irradiator (Gamma Beam GB-127, serial number IR-226; Nordion, Ottawa, Canada), with a cobalt-60 source (activity 14416 Ci), was used to irradiate groups of 1500 to 2500 male pupae in 50 mL of deionized water in 10.5 cm diameter plastic trays. Pupae received a dose of 50 Gy over a 10–12 min period as described previously, as this induces a sterility of 99.4% [36]. Irradiated pupae were then placed in adult emergence chambers at 26 ± 2 °C, for 48 h. Very few male pupae (<1%) failed to emerge as adults. The emergence chambers consisted of a 250 mL capacity plastic tub coupled to a funnel through which adult males passed to an upper chamber of 1 L capacity that was ventilated at the sides with fine nylon mesh and they had continuous access to a cotton pad placed on the lid that was moistened with 10% sucrose solution (Supplementary Figure S1). The interior walls of the adult chamber were covered with paper that was impregnated with 35 mg of fluorescent dust (BioQuip Products, Compton, CA, USA). In this way, sterile males were marked with yellow dust for the ground release and pink dust for aerial release. All males acquired fluorescent powder by this technique; unmarked males were never observed. Previous studies indicated that powder marking young males did not affect their survival or host-seeking behavior [39,40]. Emergence chambers with males were chilled at 4 ± 1 °C for 15 min in an upright laboratory refrigerator to immobilize the males as the pupal container was removed. Very few males were lost during this procedure—generally less than five individuals per container. Marked males remained in the adult container and were fed ad libitum with 10% sucrose at 26 ± 2 °C until used for field releases 3–4 days after emergence.

### 2.3. Estimation of Released Sterile Males

Forty-eight hours after irradiated pupae had been placed in emergence chambers, live and dead pupae and non-flying adults in the lower section and live and dead adult males in the upper section were counted in a randomly selected sample of 15% of the emergence devices. Any samples in which contamination by female individuals exceeded 1 in 1500 (0.067%) were returned to the plate-separation process to eliminate those females. The proportion of living adult males that emerged from a known number of irradiated pupae was then used to estimate the total number of sterile males released from each container each week. For ground releases, the estimated release was adjusted to account for males that were observed to have died and that remained in release containers following ground release. This procedure was not possible for aerial releases as all insects present in release canisters were liberated during the drone flight, so the estimates of the number of males released were based on the numbers of adult males that were present in the upper emergence chamber prior to loading the release canisters.

### 2.4. Experimental Release and Sampling

The study was initiated on 1 October 2018 and consisted of 11 weekly releases of sterile males using the ground release method (1 October to 10 December). Aerial releases began three weeks later and consisted of 8 weekly releases from 22 October to 10 December 2018. Marked sterile males were released once a week every Monday using both methods. On release days, between 06:30 and 07:00 a.m., between 15 and 18 containers, each containing approximately 1600 marked males destined for ground release, were transported from the mass-rearing facility to Hidalgo village (~5 km distance) in containers in the back of a pick-up truck. Males had continuous access to 10% sucrose solution on a cotton pad prior to release. The vehicle was parked and then four technicians placed the containers into backpacks and released the sterile males while walking through nearby streets. The pick-up truck was then moved to another street and the operation was repeated until the entire area of the village was covered. Between three and seven containers of sterile males were released in each street at distances of approximately 100–200 m. Ground releases were performed from 8:00 to 9:30 am, after which dead males in containers were counted and noted for an adjustment of actual numbers of living marked males released.

For aerial release, containers with ~1600 sterile males were chilled at 4 ± 1 °C for 20 min to immobilize the insects that were then transferred to two aluminum ventilated cages of 12 × 15 × 30 cm, (Servicios Mubarqui, Ciudad Victoria, Tamaulipas, Mexico). Loaded ventilated cages (Supplementary Figure S2) were transported from the rearing facility to Hidalgo village at 6 ± 1 °C in a portable electric cooler (Dometic CF-110, Big Prairie, OH, USA) (Supplementary Figure S3) [25]. The village was divided in two parts, with an area of 16 ha to the west and an area of 10 ha to the east. The cage assigned to the larger area contained an average of ~47,000 sterile males, whereas the cage for the smaller area contained an average of ~39,000 males for each release. Aerial releases were made using a drone (DJI Matrice 600, Shenzhen DJI Sciences and Technologies Ltd., Nanshan District, Shenzhen, China) fitted with a tubular release container placed at a 45° angle on the bottom of the drone. Chilled male mosquitoes were gently loaded into the top of a tubular release device (7.5 cm diameter × 45 cm length) which was connected to a vibratory system (Supplementary Figure S4). The vibration system was located at the bottom gate of the cylindrical container. The frequency of vibration was controlled by adjusting the power to an eccentrically balanced motor. The aperture in the gate was also controlled electronically and the combination of gate aperture and vibration allowed us to calibrate the rate of mosquito release per unit time. Sterile males were released from 8:00 to 8:30 am at a height of 50 m over the ground, which was sufficient to avoid tall obstacles such as trees and power lines. Two flights (~10 min each) departed from the center of the village on two routes in large “S” shaped flights according to pre-programmed flight plans to cover the entire inhabited area (Supplementary Figures S5 and S6). The prevailing westerly wind during aerial releases did not exceed 5 km/h (1.4 m/s). The rate of release of sterile insects was calibrated at ~80 males/second (equivalent to approximately 14 males/m at the programmed flight speed of 20 km/h). Both release methods were employed during this 8-week period.

Sampling of *Ae. aegypti* sterile males, and wild (unmarked) *Ae. aegypti* and *Ae. albopictus* was performed using BG-Sentinel traps with the proprietary BG lure, without a carbon dioxide source (Biogents AG, Regensburg, Germany) in houses distributed across the village. With the occupant’s permission, one trap was placed inside or in the backyard of each selected house in an appropriately shaded location. Each house was georeferenced using a GPS locator (Figure 1). Traps were sampled for 12 weeks between 4 October and 20 December 2018. Sampling was performed in and outside 13 houses during the first 4 weeks of the study and this was increased to 16 houses for weeks 5 to 12. Overall, half of the traps were placed inside houses and half were located outside. On occasion, it was not possible to gain access to a selected house, which resulted in one less trap sampled in a particular week (Table 1). Traps were activated 48 h after each release of sterile males and were operated for a 24 h period, after which trap samples were collected and taken to the laboratory for identification. Trap positions in the selected houses were changed every week to avoid positional biases.

The air temperature and relative humidity at the moment of male releases (08:00–09:30 a.m.), and at the moment of sampling trap catches (08:30 a.m.–12:00 p.m.) were measured using a digital thermometer-hygrometer (HTC-1, Ace Instruments, Hyderabad, India). Precipitation and mean daily temperature records were obtained from a weather station located 6.8 km from the village.

### 2.5. Laboratory Processing of Samples

Adult mosquitoes from each BG-Sentinel trap were stored in separate containers. Preliminary studies showed that this procedure did not result in the transfer of fluorescent powder among marked insects, or from marked to unmarked insects. All collected mosquitoes were killed by freezing, sexed and morphologically identified to genus and species using a stereomicroscope and an identification key [41]. All *Ae. aegypti* males were examined under UV light for the presence of fluorescent powder [42]. The weekly recaptures of *Ae. aegypti* sterile males were calculated as the mean number and percentage of marked males found in traps compared to the number of males released that week by each method. We assumed that interference from marked males that survived from the previous week’s releases would be negligible. Similarly, the prevalence of traps that captured at least one marked male in each weekly sample was calculated based on the total number of traps sampled.

### 2.6. Statistical Analyses

The number of recaptured marked males in relation to the number of males released by each method was analyzed by fitting a generalized linear mixed model for repeated measures with a binomial response. Over-dispersion was taken into account by introducing an extra parameter in the variance-mean relationship [43]. Numbers of traps with recaptured marked males in relation to the number of traps without recapture of marked males by each method were analyzed by fitting a generalized linear mixed model for repeated measures with a negative binomial response specified and the release method as a fixed factor. The number of sterile males recaptured in each method was spatially analyzed by interpolating the recapture spatial data, converting these into distances using the geographical coordinates of each trap and by using these values for non-parametric nearest neighbor interpolation. All analyses were conducted in R v.4.0.5 [44].

## 3. Results

The mean air temperature at the moment of the male releases with the aerial and ground methods was 26.3 ± 0.4 °C (range 24.5–28.7 °C) and 27.9 ± 0.3 °C (range 24.5–31.6 ℃), respectively, whereas mean relative humidity was 87.8 ± 1.9% (range 76–99%) and 81.2 ± 1.4% (range 65–99%), respectively. The mean air temperature at the moment of trap sampling was 31.9 ± 0.2 °C (range 25.4–40.1 °C) and the mean relative humidity was 63.6 ± 0.9% (range 39–94%). Mean weekly precipitation during the study period was 54.6 ± 19.4 mm (range 0–157 mm).

Overall, a total of 1,633,096 of *Ae. aegypti* sterile males were released during the 11-week study, at an average of 148,463 ± 16,054 sterile males/week (Table 1). During the 8 weeks involving both release methods, a weekly average of 85,117 ± 6457 males were released at ground level and 86,724 ± 6474 males were released using the aerial drone method.

A total of 1003 *Ae. aegypti* marked males from both release methods were recaptured during 12 weeks of trap sampling. In addition, 306 wild *Ae. aegypti* (168 males, 138 females), 10 *Ae. albopictus* (1 male, 9 females) and 199 *Culex* spp. (57 males, 142 females) were also collected from traps). *Culex quinquefasciatus* was the most abundant *Culex* species, but was not considered further.

**Table 1:** Number of sterile male *Aedes aegypti* released using ground and aerial methods and captures of sterile and wild males in BG-traps in Hidalgo village during a 12-week period (October–December 2018).

	Number of *Ae. aegypti* Sterile Males Released by Each Method			Sterile Males Recaptured from Ground Releases		Sterile Males Recaptured from Aerial Releases		Wild Males of *Ae. aegypti* Collected in Traps	
Week	Ground	Aerial	No. Traps Sampled	Total	Mean Number/Trap ± SE (%) ^1^	Total	Mean Number/Trap ± SE (%) ^1^	Total	Mean/Trap ± SE
1	79,000	0	12	34	2.83 ± 1.8 (0.04)	-	-	8	0.7 ± 0.4
2	51,502	0	10	8	0.80 ± 0.3 (0.02)	-	-	0	0.0 ± 0.0
3	109,238	0	12	21	1.75 ± 1.1 (0.02)	-	-	42	3.5 ± 1.7
4	68,579	68,618	13	61	4.7 ± 1.9 (0.09)	24	1.8 ± 0.7 (0.03)	6	0.5 ± 0.2
5	88,701	88,932	16	251	15.7 ± 9.7 (0.28)	116	7.3 ± 4.4 (0.13)	8	0.5 ± 0.3
6	74,751	74,751	15	58	3.9 ± 1.6 (0.08)	39	2.6 ± 2.5 (0.05)	15	1.0 ± 0.6
7	111,354	113,350	15	93	6.2 ± 3.3 (0.08)	50	3.3 ± 1.9 (0.04)	20	1.3 ± 0.9
8	60,808	62,615	16	44	2.8 ± 1.3 (0.07)	13	0.8 ± 0.5 (0.02)	17	1.1 ± 0.4
9	109,716	110,616	16	34	2.1 ± 0.7 (0.03)	44	2.8 ± 2.2 (0.04)	9	0.6 ± 0.3
10	78,424	85,320	15	16	1.1 ± 0.3 (0.02)	8	0.5 ± 0.3 (0.01)	31	2.1 ± 0.9
11	88,600	89,591	16	64	4.0 ± 1.5 (0.07)	22	1.4 ± 0.4 (0.02)	7	0.4 ± 0.3
12	0	0	16	1	0.1 ± 0.1 (-)	2	0.1 ± 0.1 (-)	5	0.3 ± 0.2
Total	920,673	693,793		685		318		168	

^1^ Values in parentheses are percentages calculated from the weekly numbers of sterile male mosquitoes captured and released. Insects were captured over a 24 h period using traps that were activated 48 h after the release of marked irradiated males.

For the 8-week period during which both release methods were employed, 937 sterile males of *Ae. aegypti* were recaptured, of which 621 were from the ground releases, and 316 originated from aerial releases (Table 1). The weekly numbers of marked sterile males captured from ground release method was significantly higher (mean = 5.1 ± 1.4/males/trap, range 1.1–15.7 males/trap) than those captured from the aerial release method (mean = 2.6 ± 0.8 males/trap, range 0.5–7.3 males/trap) in the samples taken at 4–11 weeks (repeated measures GLM, *χ*^2^ = 4.6, *df* = 1, *p* < 0.05). These recapture rates represent 0.02–0.28% of each week’s release by the ground release method compared to 0.01–0.13% of each week’s release by the aerial drone method.

**Figure 2:**
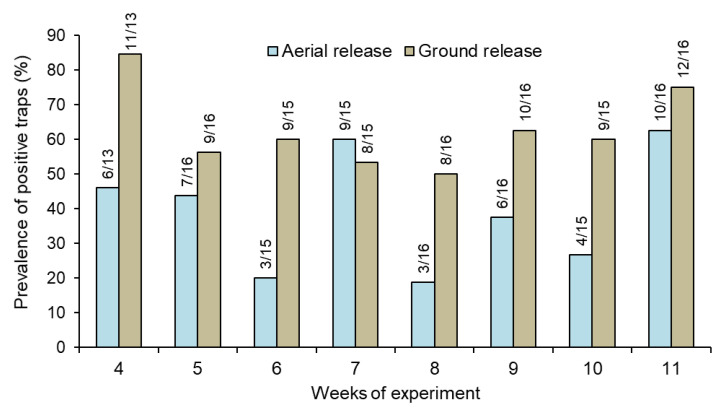
Prevalence (%) of traps that captured at least one sterile male *Aedes aegypti* released through ground and aerial in houses in Hidalgo village. Numbers above columns indicate the number of positive traps out of the total number of traps sampled each week (see Table 1 for the weekly number of traps sampled).

The percentage of traps that captured at least one sterile male each week was also significantly higher in the ground release (range 50–85%) compared to the aerial release (range 19–63%) treatment (repeated measures GLM, *χ*^2^ = 13.0, *df* = 1, *p* < 0.01) (Figure 2; Supplementary Figure S7).

**Figure 3:**
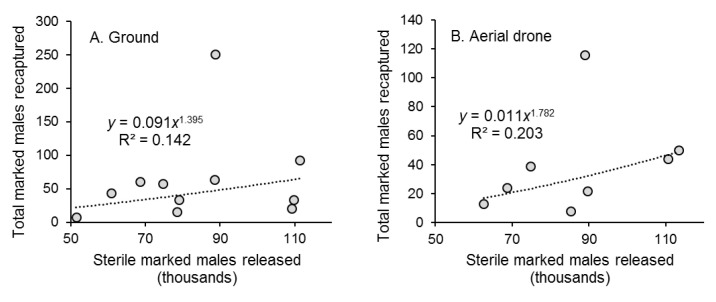
Correlation between the weekly number of marked sterile males of *Aedes aegypti* released (in thousands) by the (**A**) ground release method and (**B**) aerial drone method and the total number of marked males recaptured each week during 11 weeks of ground releases and 8 weeks of aerial releases in Hidalgo village, southern Mexico. Each graph shows the correlation equation and coefficient of determination (R^2^).

There was a weak correlation between the weekly number of marked males released and the number captured in traps for both ground and aerial release methods (Figure 3A,B). There was no correlation between the number of recaptured marked males and mean air temperature, or total precipitation recorded at the nearby meteorological station during the 72 h period post-release (data not shown).

**Figure 4:**
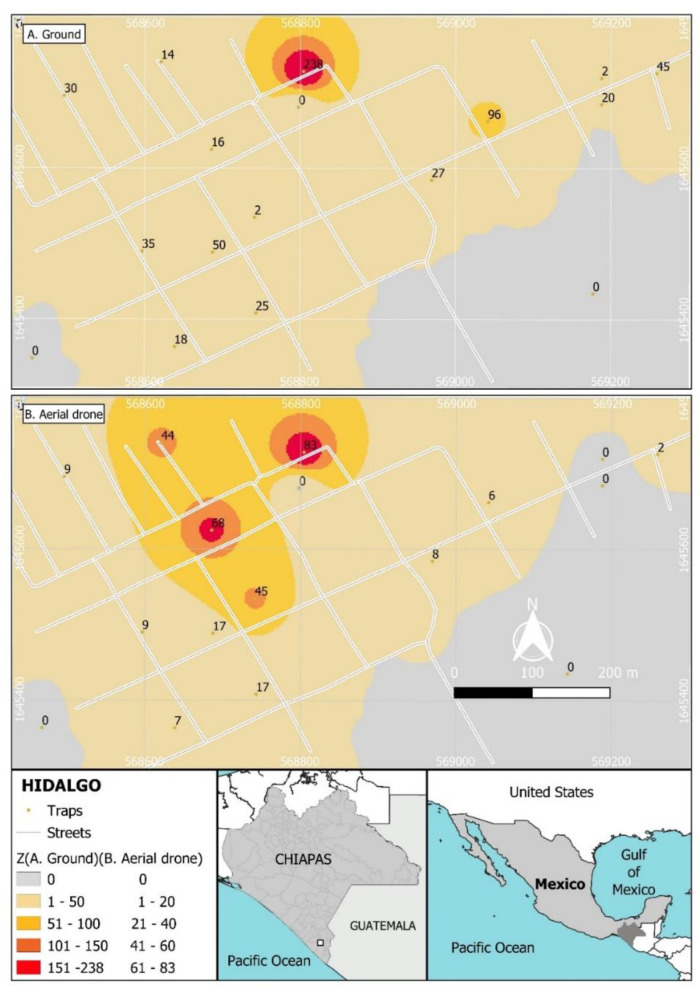
Spatial distribution of recaptures of sterile males of *Aedes aegypti* released through two methods in the Hidalgo village: (**A**) recaptures from ground release, (**B**) recaptures from aerial release. Colors indicate total recaptures ranging from grey to red (50–200 insects following ground release in (**A**); 20–80 insects following aerial release in (**B**)). Values indicate the total numbers of sterile males captured during the 8-week period of both releases.

Unexpectedly, the recapture of the males from the ground release was spatially aggregated in the North of the village (Figure 4A), whereas recapture following aerial release was most frequent in the North and central area of the village (Figure 4B).

Overall, based on the 24 h trap capture period, the ground releases resulted in a sterile: wild male ratio of 4:1 over the 11-week trial (based on a total of 684 marked males vs. 168 wild males), compared to a 5.5:1 ratio during the period of both releases (based on a total of 621 marked males and 113 wild males captured between week 4 and week 11). In contrast, the sterile: wild ratio of males from aerial releases was 2.8:1 over the 8-week period of both releases (based on a total of 316 marked males vs. 113 wild males) (Table 1). Interestingly, the final sample taken at week 12, in which no sterile males were released and only one or two marked males were recaptured from the previous week’s releases (Table 1), indicated that the numbers of sterile males captured each week were unlikely to be affected by the previous week’s releases during the 8-week period of both release methods.

## 4. Discussion

Ground-based and aerial drone releases of marked sterile males of *Ae. aegypti* were compared by analyzing the presence of marked males in BG-Sentinel traps in a rural village in the southern of Chiapas, Mexico. Despite being released in almost equal numbers during the 8-week period of both releases, sterile males released at ground level were captured twice as frequently as those released by aerial drone. Ground-released males were also captured in a significantly higher proportion of the available traps placed throughout the village (Figure 2).

These findings are likely to have been influenced by the proximity of street-level ground-based releases to houses, whereas aerial released males likely had to fly across greater distances to reach a house containing a trap (Figure 4A,B). However, in both cases, a single trap located in the northern section of the village captured markedly higher numbers of marked males than any other trap. This trap also had the third highest capture of wild *Ae. aegypti* males. It is unclear why this trap was so attractive to males, although we note that it was just 25 m away from the village Junior-high school, which may have provided attractive olfactory stimuli to the dispersing mosquitoes. The relatively low sterile: wild male ratios observed in our study (Table 1) were likely due to a combination of the significant immigration of wild males from adjacent untreated areas and emigration of sterile males to surrounding areas, given the relatively small area (26 ha) over which sterile mosquitoes were released. A previous study that compared captures of *Ae. aegypti* irradiated males released at ground level or by aerial drone at altitudes of 50 or 100 m also reported higher captures of ground released mosquitoes compared to drone-released males and the aggregation of males in certain localities in the study area, presumably in response to climatic conditions or the presence of conspecific females [26]. 

Reciprocal marking was not performed in our study as we wanted to avoid possible confusion in the identification of each type of marked mosquito during weekly sampling. We have no evidence to indicate or refute the idea that different colors may have affected mosquito survival or trap responses following releases, although a previous study found no evidence for differential survival in *Ae. aegypti* females marked with one of seven different fluorescent powders [40]. We consider it unlikely that mosquito captures would be significantly affected by releases in previous weeks as most *Ae. aegypti* do not survive for more than a few days under field conditions at 20–30 °C [45,46]. The observation that on week 12 no marked males were released and trap captures were near zero (0.1 males/trap, Table 1) supports the assumption that insects released the previous week had a negligible effect on weekly trap captures in our study.

The ground release method had several disadvantages for scaling up and implementation in an area-wide SIT-based program. The release time was 4.5-fold longer for ground releases compared with the aerial release method. In this study, ground release required five technicians (four performing releases and one to drive the truck) and an average of 1.5 h to release males over an area of 26 hectares. In contrast, drone-based aerial releases could be performed by two technicians and the entire village could be treated in a 20 min period. Ground releases over larger areas would therefore involve a larger labor pool and increased costs [26], and the risk of higher insect mortality prior to release as daytime temperatures rise quickly after 09:00 am in tropical regions, such as southern Mexico. However, the purchase, routine maintenance and operational costs of aerial drones equipped with mosquito release devices can be high and should be considered against the costs of ground-based operations.

The use of technological tools could greatly assist the deployment of area-wide SIT-based programs [25]. Drones have clear potential for the release of sterile males of different disease vectors in rural areas, towns and cities [26]. Drones can also improve the surveillance of breeding sites on roofs and backyards that are not easily accessible to ground surveillance teams [47]. Aerial release also has the advantage of distributing sterile individuals more evenly than ground releases and allows for the rapid treatment of impenetrable areas such as dense forest, steep canyons, fenced-off properties or densely planted crops such as sugarcane that technicians cannot access by walking. The aerial release method also resulted in fewer aggregations of males in and around houses that disturbed residents [26]. Set against this background, the use of an aerial drone is contingent on climatic conditions, particularly at higher windspeeds, whereas ground-based releases do not face such limitations, except in the case of heavy rainfall.

The decision to open BG traps 48 h after mosquito releases represented a tradeoff between allowing mosquitoes time to adapt to field conditions and disperse over the area of the village, set against mosquito dispersal away from the village and the reduced survival of sterile males under natural conditions [30,40]. Under laboratory conditions, 50 Gy-irradiated adult males of *Ae. aegypti* had a median survival time of 46 days compared to 54 days in non-irradiated males [36]. In a different field study, the survival of ground-released *Ae. aegypti* males was similar to that of drone-released males at altitudes of 50 or 100 m [26]. It seems unlikely, therefore, that irradiated males would have experienced particularly poor survival during the three-day interval between insect release and trap sampling. Nevertheless, opening the traps earlier may have resulted in increased numbers of captured males from both release methods.

The lower numbers of sterile males trapped following aerial releases may also have been affected by mortality or physical compaction during handling and chilling prior to the transport of sterile males. In the present study, all males were chilled at 4 °C for 15 min so as to collect marked males from the emergence chambers, but males destined for drone release were subsequently chilled to 4 °C for 20 min to load the drone emergence tubes. Insects were also transported at 6 °C from the rearing facility to Hidalgo village, a trip that lasted 10–15 min [27]. It was not possible to assess the mortality of sterile males after loading the drone in the village at the start of the release flights. Previous laboratory studies failed to detect the negative effects on survival in non-irradiated males of *Anopheles arabiensis* up to 14 days after being exposed to a chilling of 4–10 °C for up to 24 h [27]. Similarly, the survival of males of *Ae. aegypti* was not significantly affected by exposure to temperatures of 4–10 °C for a period of 2 to 8 h [28,29] or a temperature of 7 °C for 24 h [48]. However, the flight ability, mating success and insemination capacity of *Ae. aegypti* males decreased when insects were chilled at a temperature of below 8 °C [28]. In contrast, for *Ae. albopictus* males, conflicting reports indicate that a 1 h exposure to 2–10 °C reduced survival [29], whereas 3 h exposure to 5–10 °C did not reduce male survival [49].

The compaction of insects during transport and aerial release carries risks of physical damage that can affect the dispersal and competitiveness of sterile males [26,48,50]. Compaction in a column of immobilized insects poses the highest risk to individuals at the base of the column, due to the weight of conspecific insects above. In this study, the ventilated cages contained between 39 and 47 thousand sterile males placed horizontally in the portable refrigerator to reduce compaction. Moreover, the 45° position of the release container placed on the drone and the short time spent within the release tube should have helped to minimize the risk of compaction of sterile males in our study. Previous studies indicate that the compaction and the chilling process during the immobilization of mosquitoes can decrease male survival, depending on the chilling temperatures, level of compaction, confinement time and the species of mosquito. For example, the survival and insemination capacity of immobilized males of *Ae. aegypti* at 4–14 °C was found to be significantly affected by increased compaction [26,28,48], whereas immobilized males of *Ae. albopictus* or *Anopheles arabiensis* were not seriously affected by compaction at temperatures between 6 °C and 12 °C [27,49]. However, the survival of *Ae. albopictus* chilled males decreased when containers were stacked vertically and males were compacted at depths of up to 8 cm [49].

A detailed study on the performance of irradiated males of *Ae. aegypti* released from an aerial drone in Brazil demonstrated that drone releases resulted in homogenous coverage of a rural village and irradiated males aggregated at the same sites as wild males for mating [26]. Males were released from an insulated canister mounted over a rotating cylinder with indentations that facilitated the dosing and ejection of mosquitoes. The study focused on the efficiency and correct function of the drone-mounted insect release mechanism and the temperature and insect loading conditions required to optimize the release, dispersal, survival and sexual competitivity of irradiated males. Compared to the study in Brazil, our study differed in the vibratory mechanism of insect release, the duration of the study and type of flight paths used to release insects, and the climatic conditions in our high humidity region compared to the semi-arid conditions of the Brazilian study [26].

Another issue of concern is that of the transfer of chilled insects in the warm humid conditions that are common in tropical regions, resulting in the rapid appearance of condensation droplets on the sides of ventilated cages that can trap or clump delicate insects such as mosquitoes [26]. This seems difficult to avoid, but working quickly to release insects may reduce exposure to condensation. We observed small numbers of sterile males trapped by moisture condensation, particularly in the transport containers, although as the moisture dissipated, some of them recovered and flew away. Condensation might be avoided by covering the metal walls of the transport containers with filter paper or a similar absorbent material on the container walls.

As *Ae. aegypti* is well adapted to anthropogenic conditions [51,52], it appears that aggregations of sterile males were formed following ground-based releases close to houses. This generated discomfort for some villagers, who complained of clouds of mosquitoes that stuck to their skin in the high humidity conditions in the village. High numbers of male mosquitoes inside houses also bothered inhabitants performing housework or other domestic activities, particularly in the afternoon and evening periods. Field technicians always engaged with villagers that expressed concerns over mosquito releases to explain the innocuous nature of non-biting male mosquitoes and to provide reassurance on the safety of the sterile insects. Interestingly, a recent study found that many households in this area had knowledge of the origin and transmission of mosquito-borne arboviruses, but very few adopted practices to reduce the risk of these diseases [53]. 

Several factors may have affected the dispersal of the sterile males released by the drone in this study. To improve male survival and dispersal, we suggest that males destined for aerial release be chilled at a slightly higher temperature, e.g., 6 °C instead of 4 °C and to use an 8 °C temperature for transport to the field site. The transport containers and release container could be modified to reduce the compaction and risk of physical injury to males. One option would be to place fewer males in each container, combined with a greater number of drone flights as they can be achieved in just a few minutes.

## 5. Conclusions

Ground-level releases of sterile males resulted in higher numbers of recaptures, and a greater presence of marked males among the available traps in the village, but required the labor of five field technicians. Drone-mediated aerial releases were markedly faster than ground-based releases and required just two technicians. Drone-mediated releases did not result in large aggregations of males and appeared to be more acceptable to the village inhabitants. Handling and transport improvements should be evaluated to reduce the compaction of males and improve the efficiency of aerial releases for area-wide SIT-based vector suppression programs.

## Data Availability

All the data presented in this study are available in the file Supplementary Data File S1.xlxs.

## Supplementary Materials

The following supporting information can be downloaded at: https://www.mdpi.com/article/10.3390/insects13040347/s1, Figure S1: Sterile male emergence chamber; Figure S2: Ventilated cage used for the transport of *Aedes aegypti* sterile males to the release site; Figure S3: Portable refrigeration unit used to transport *Aedes aegypti* sterile males from the production laboratory to the field site; Figure S4: Loading sterile males of *Aedes aegypti* into the release device attached to the aerial drone immediately prior to release; Figure S5: Aerial release of *Aedes aegypti* sterile males from the tube device attached to the drone in flight. Figure S6: Flight plans at the Hidalgo village used for the aerial release of *Aedes aegypti* sterile males using a drone that flew two routes. Figure S7: Mean proportion of positive traps that captured at least one sterile male *Aedes aegypti* released through the ground and aerial drone method in Hidalgo village.
