# Supplementary material for: Comparison of Ground Release and Drone-Mediated Aerial Release of Aedes aegypti Sterile Males in Southern Mexico: Efficacy and Challenges
Source: Insects. 2022 Mar 31;13(4):347. doi: 10.3390/insects13040347 (PMC9025923; doi:10.3390/insects13040347)
Supplement: Supplementary file 1 [file insects-13-00347-s001.zip › insects-1608843-Supplementary Material Figures.pdf]

SUPPLEMENTAL MATERIAL

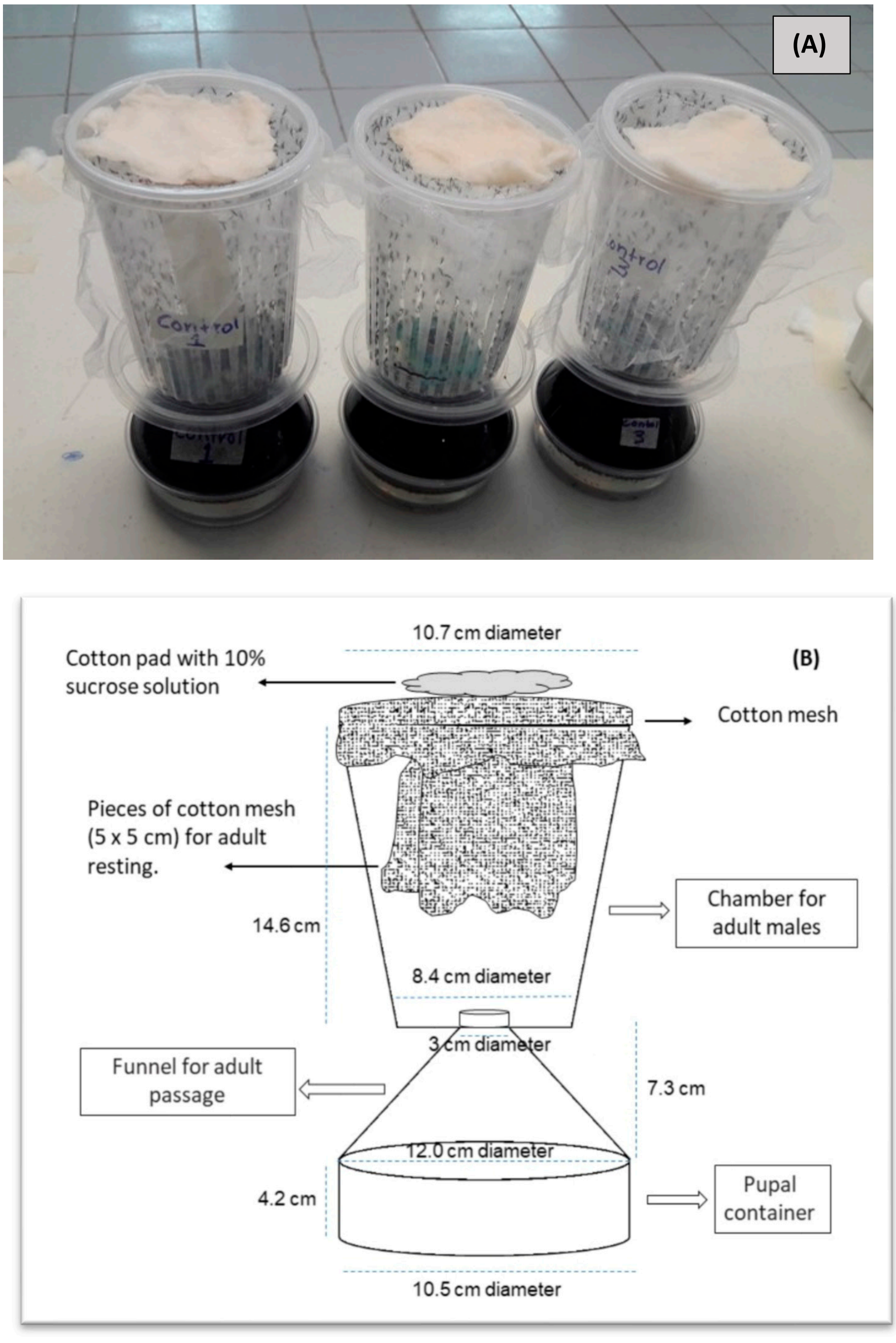

Supplementary Figure S1. Sterile male emergence chamber. (A) Picture of emergence chambers for adult sterile males. (B) Design and dimensions of the emergence chamber with its different components.

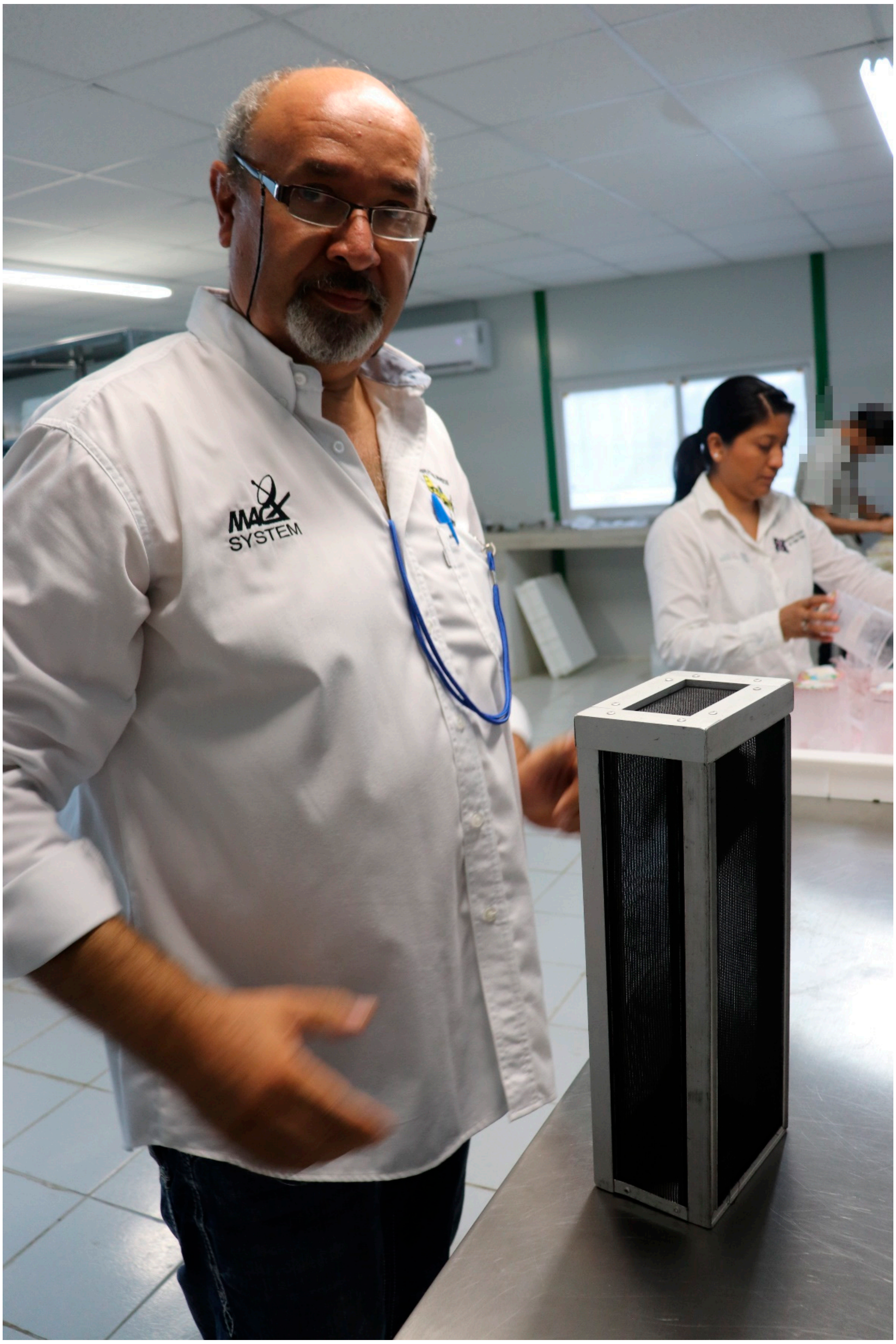

Supplementary Figure S2. Ventilated cage used for the transport of *Aedes aegypti* sterile males to the release site.

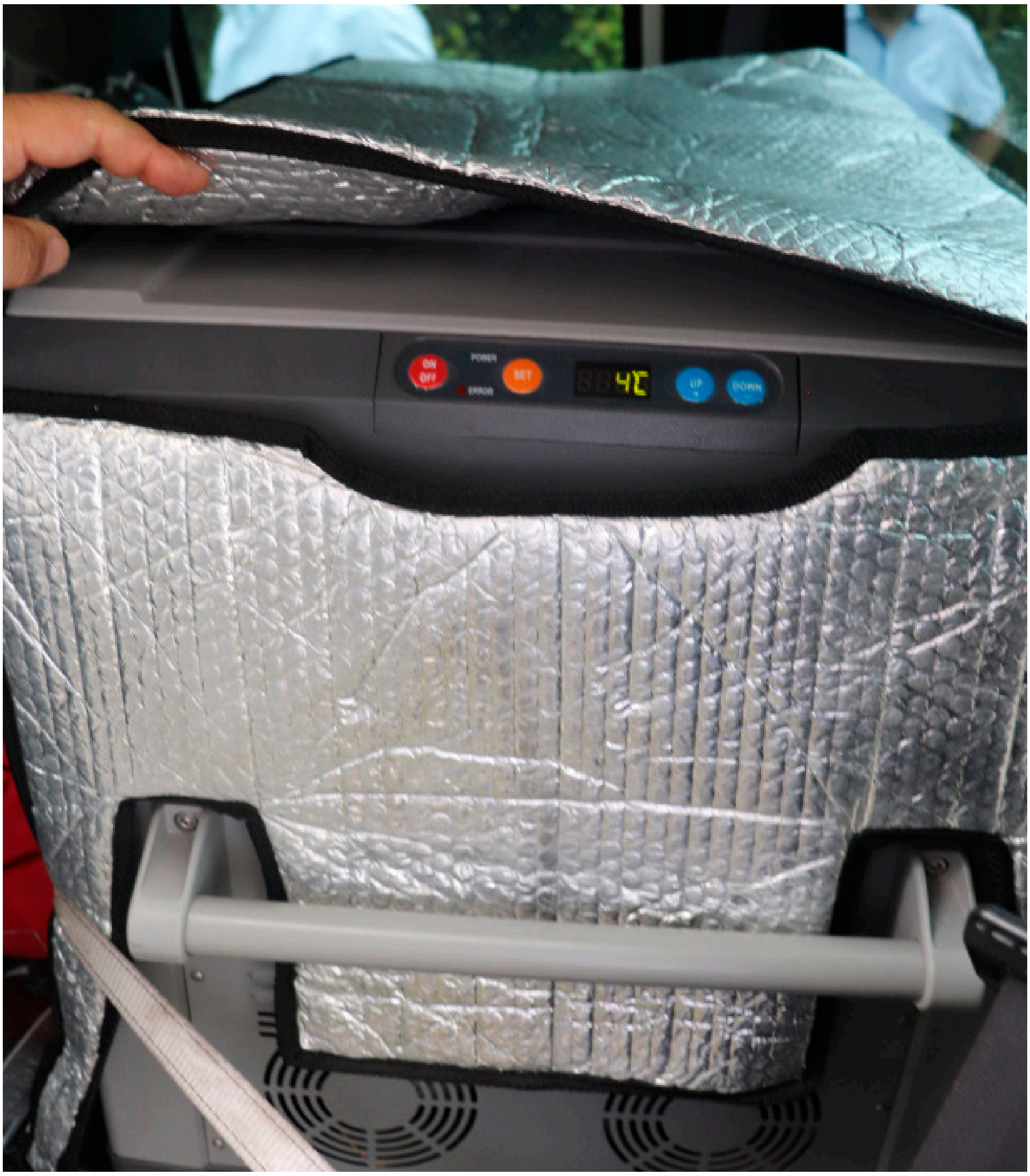

Supplementary Figure S3. Portable refrigeration unit used to transport *Aedes aegypti* sterile males from the production laboratory to the field site.

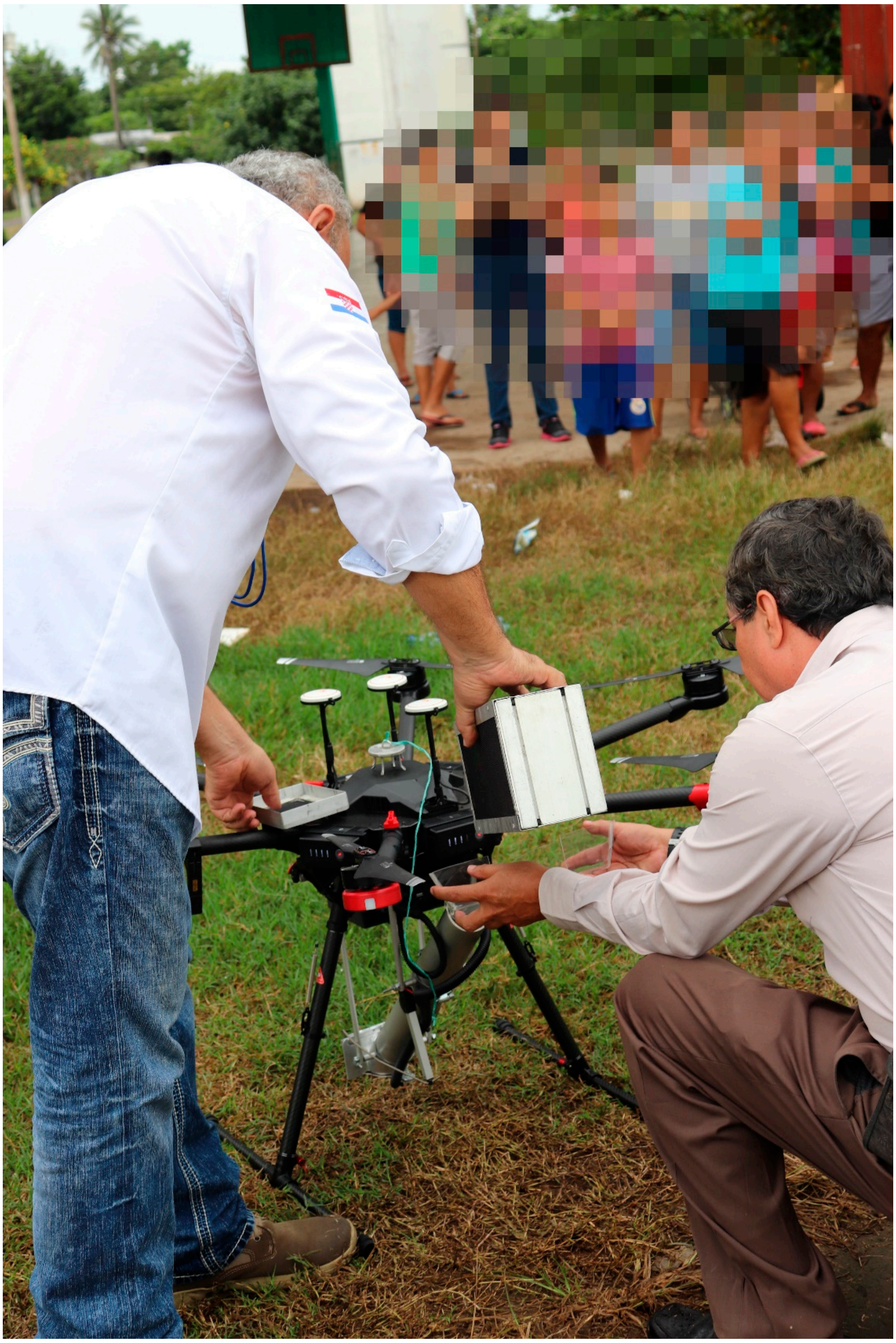

Supplementary Figure S4. Loading sterile males of *Aedes aegypti* into the release device attached to the aerial drone immediately prior to release.

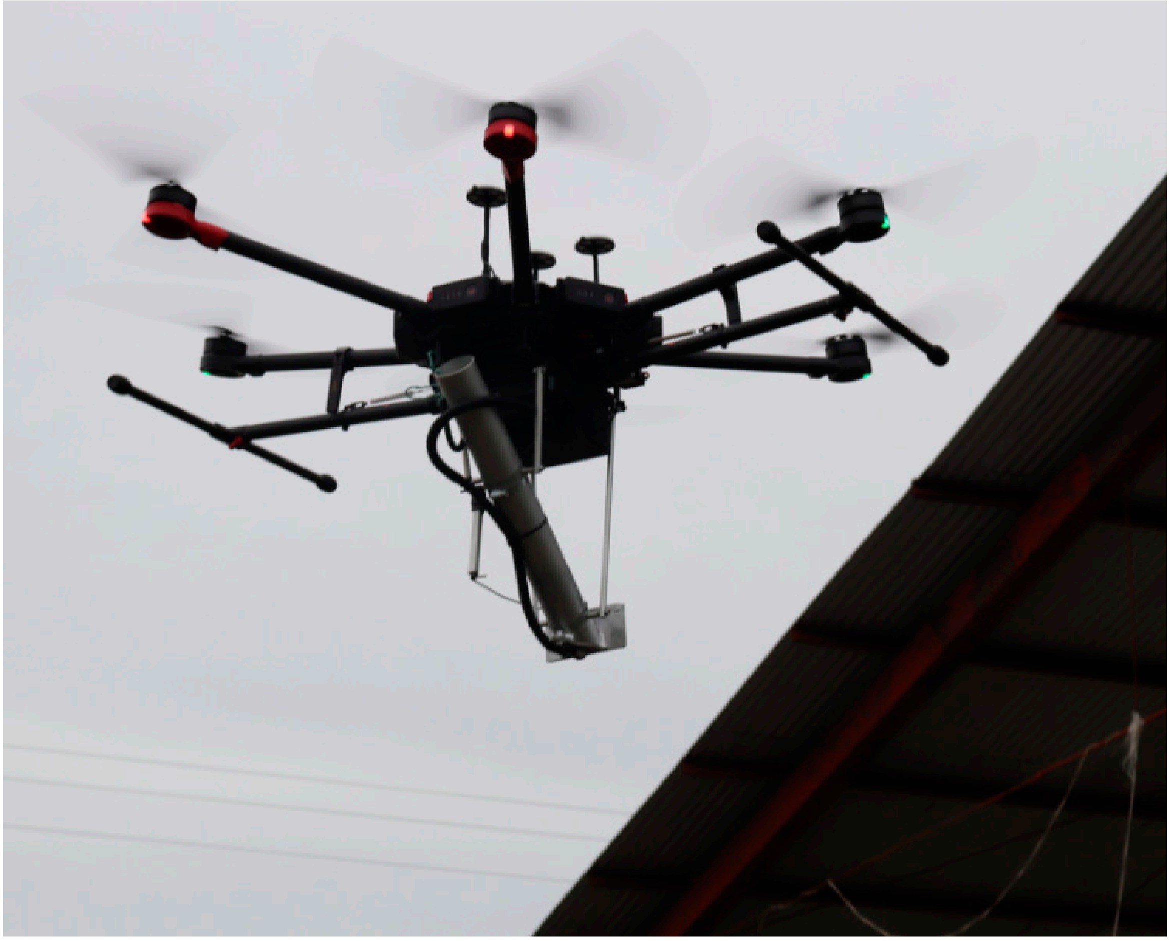

Supplementary Figure S5. Aerial release of *Aedes aegypti* sterile males from the tube device attached to the drone in flight.

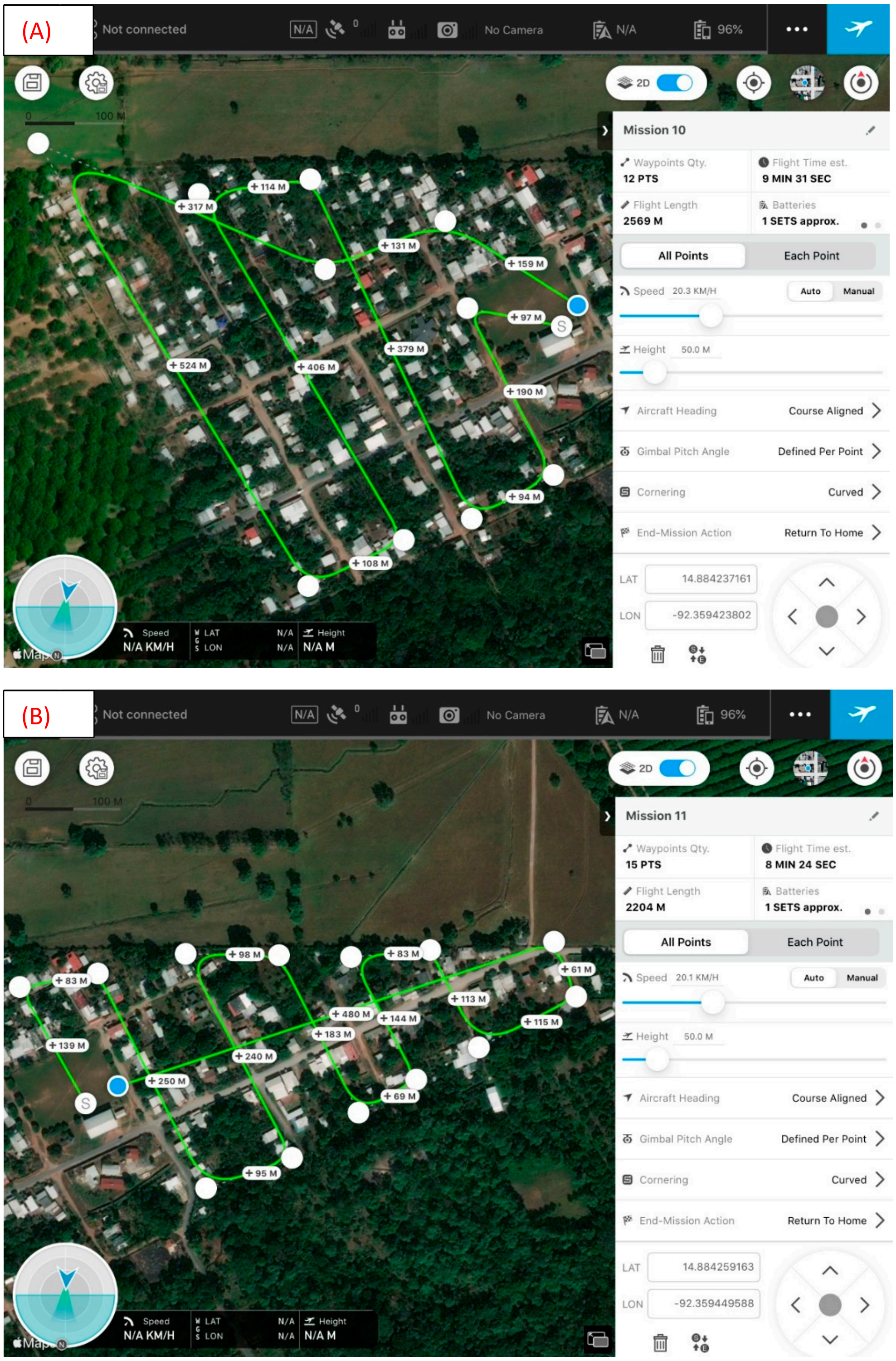

Supplementary Figure S6. Flight plans at the Hidalgo village used for the aerial release of *Aedes aegypti* sterile males using a drone that flew two routes: (A) first route over the West side of the village, (B) second route over the East side of the village. Letter "S" within white dot indicates the starting point of the flight. White

dots indicate points on the flight path where the drone was programmed to change course. Blue dot indicates the end of mission or landing point. Values in meters indicate distances between white dots.

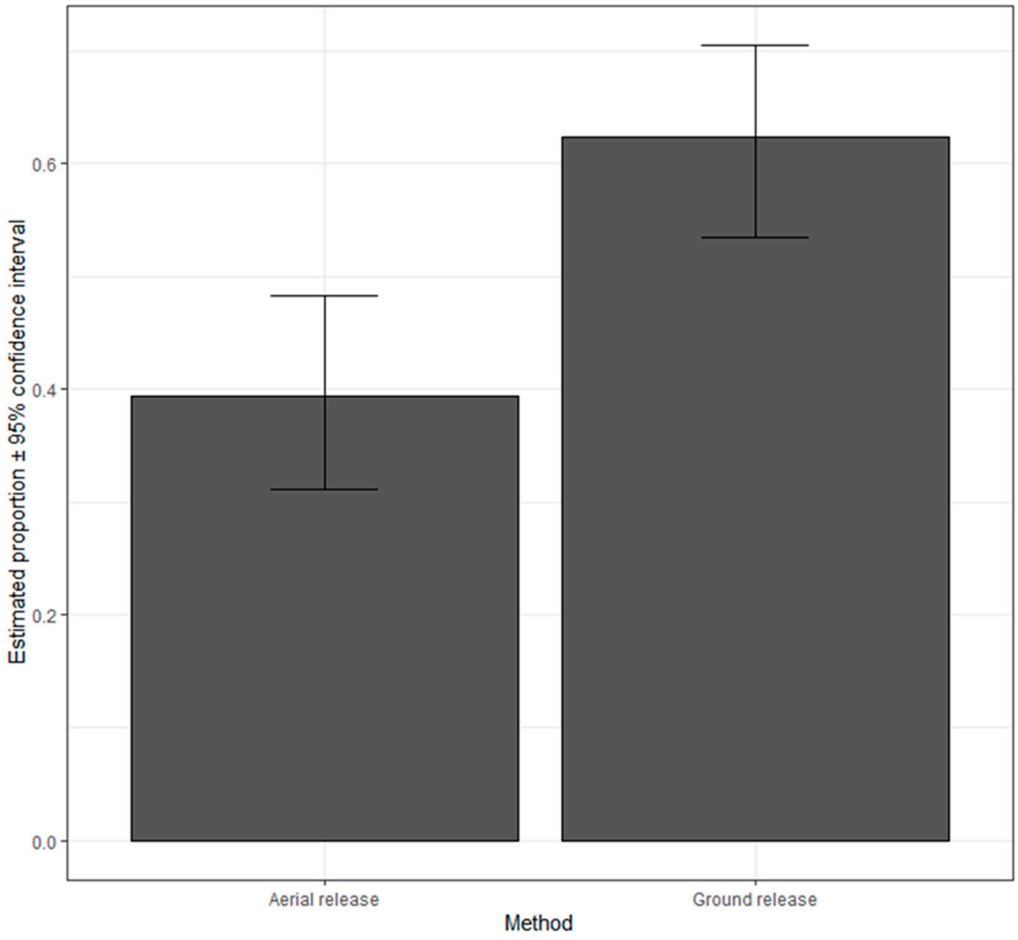

Supplementary Figure S7. Mean proportion of positive traps that captured at least one sterile male *Aedes aegypti* released through the ground and aerial drone method in Hidalgo village. Means were estimated over the eight week period for which insects were released by both methods. Error bars indicate 95% confidence interval.
